# TB Mobile: a mobile app for anti-tuberculosis molecules with known targets

**DOI:** 10.1186/1758-2946-5-13

**Published:** 2013-03-06

**Authors:** Sean Ekins, Alex M Clark, Malabika Sarker

**Affiliations:** 1 Collaborative Drug Discovery, 1633 Bayshore Highway, Suite 342, Burlingame, CA 94010, USA; 2 Collaborations in Chemistry, 5616 Hilltop Needmore Road, Fuquay-Varina, NC 27526, USA; 3 Molecular Materials Informatics, 1900 St. Jacques #302, Montreal, Quebec, H3J 2S1, Canada; 4 SRI International, 333 Ravenswood Avenue, Menlo Park, CA, 94025, USA

**Keywords:** Collaborative drug discovery tuberculosis database, Drug discovery, Mobile applications, *Mycobacterium tuberculosis*, Tuberculosis, TB Mobile

## Abstract

**Background:**

An increasing number of researchers are focused on strategies for developing inhibitors of *Mycobacterium tuberculosis* (*Mtb*) as tuberculosis (TB) drugs.

**Results:**

In order to learn from prior work we have collated information on molecules screened versus *Mtb* and their targets which has been made available in the Collaborative Drug Discovery (CDD) database. This dataset contains published data on target, essentiality, links to PubMed, TBDB, TBCyc (which provides a pathway-based visualization of the entire cellular biochemical network) and human homolog information. The development of mobile cheminformatics apps could lower the barrier to drug discovery and promote collaboration. Therefore we have used this set of over 700 molecules screened versus *Mtb* and their targets to create a free mobile app (TB Mobile) that displays molecule structures and links to the bioinformatics data. By input of a molecular structures and performing a similarity search within the app we can infer potential targets or search by targets to retrieve compounds known to be active.

**Conclusions:**

TB Mobile may assist researchers as part of their workflow in identifying potential targets for hits generated from phenotypic screening and in prioritizing them for further follow-up. The app is designed to lower the barriers to accessing this information, so that all researchers with an interest in combatting this deadly disease can use it freely to the benefit of their own efforts.

## Background

Modern drug discovery must be more time- and cost-efficient in discovering novel therapeutics. These challenges are felt even more significantly in the search for neglected disease treatments. A prime example is tuberculosis (TB), caused by *Mycobacterium tuberculosis* (*Mtb*), which infects approximately one-third of the world’s population and results in 1.7–1.8 million deaths annually [1]. New drugs active against *Mtb* are urgently needed to combat a pandemic heavily affected by resistance to available therapies and co-infection with HIV/AIDS [2]. The pipeline for TB therapeutics had not produced a new approved drug in over 40 years [3,4]. Only a tiny fraction of TB targets have been addressed with approved drugs and recent testing has targeted additional proteins but this has yet to result in a drug besides bedaquiline for multidrug resistant TB [5,6]. This is a pattern observed for other antibacterial targets reflecting the difficulty of target-based high-throughput screening [7]. In recent years there has been an increase in the efforts around high throughput screening (HTS) for *Mtb*, in order to find compounds as therapeutics against TB [8-12] at a cost of millions of dollars, with resultant low single-digit (or less) hit rates [8,9,13,14]. Whole cell screening, however does not provide any information on the targets for the hits that result and this therefore entails costly follow up. In many cases such target identification is unable to identify one or more target.

While there have been studies that evaluate the role of particular *Mtb* genes and define their potential as targets for new drugs [15] there have been few efforts to predict targets for hits coming from whole cell screening. Various TB-related databases [16] are available that cover diverse areas of TB research like genomes, pathway maps, phylogenetic trees, active compounds, large-scale screening data, resistance-associated mutations, targets, comparative analysis and gene expression data. Pipelines for bioinformatics processes such as target identification in TB (e.g. targetTB [17]) have also been suggested. We created a collection of >700 molecules with *Mtb* target/s along with published data on the target, essentiality, links to literature (PubMed), genes (tbdb.org), pathways (TBCyc, which provides a pathway-based visualization of the entire cellular biochemical network) and human homolog information [18] collated in the course of a previous study [19]. This dataset was made available in the Collaborative Drug Discovery (CDD) database [18].

As mobile devices such as smartphones and tablet computers have seen rapid uptake in recent years and the associated app stores include a growing number of chemistry software apps [20], making data available as an app may help reach a wider audience. These mobile apps generally perform one or two functions and can be thought of as individually packaged features rather than the relatively heavyweight programs commonly used in desktop computing. However, such apps *can* use data interchange and be used in the workflow to increase the productivity of chemists [21,22]. Mobile apps for chemistry are a nascent area to delivering or "appifying" data and may be disruptive to many currently used paradigms for presenting information and for education [23]. A recent example is the Green Solvents mobile App which took data collected on solvents and delivered it as a free look-up tool to help in solvent selection [24].

The recent collation of molecules screened versus *Mtb* and their targets could help in the task of suggesting potential targets for HTS hits [19]. This data was used to create the TB Mobile app that displays molecule structures and links to the bioinformatics data. By input of a molecule structure and performing a similarity search one can infer potential targets or search by targets to retrieve compounds known to be active. The app also has filters to limit the visible molecules by target name, pathway name, essentiality and human ortholog. We now describe TB Mobile [25,26] and its potential applications.

## Methods

### Dataset curation

The process of dataset curation can be broken down into several steps.

1. Identification of essential *in vivo* enzymes of *Mtb* involved intensive literature mining and manual curation, to extract all the genes essential for *Mtb* growth *in vivo* across species [27-31].

2. Homolog information was collated from other studies [28,32].

3. Collection of metabolic pathway information involved using TB database (TBDB) [33,34].

4. Identifying molecules and drugs with known or predicted targets [35] involved searching the CDD databases for manually curated data. The structures and data were exported for combination with the other data.

5. All data were combined with URL links to literature and TBDB [33,34] and deposited in the CDD database [18].

### TB Mobile app software development

The iOS app was build using the Objective-C programming language, with the API provided by Apple for native iOS development, while the Android app was built using Java and the standard Android API. The apps have almost identical functionality, with minor aesthetic differences due to the platform. Both of these apps are linked with *MMDSLib*, which is available separately for both platforms, and provides the core functionality for a number of cheminformatics apps, such as the Mobile Molecular DataSheet (MMDS) [36].

The TB related information was entered manually, and is bundled as part of the app resources. The TB Mobile app was developed as described above then made available on iTunes [25] as a free app for the iPhone, iPod and iPad platforms. The Android version is available free of charge on Google Play [26].

### TB Mobile app software application

The TB Mobile app uses molecule structures grouped as the primary point of entry. These molecules are listed with the targets. The user can use the swiping gesture to scroll through all the solvents, then tap on a molecule of interest. This opens a box which lists the molecule name, CDD number, and resistance information. A second box lists the target (Rv number), a link to TBDB, homolog information, essentiality information, pathway information and gene links to PubMed. Links out to open in other mobile apps like ChemSpider [37], the Mobile Reagents app [38] and MMDS etc. [39] are also provided.

### Similarity searching in TB Mobile app

Similarity comparisons are done by computing simple typed graph fingerprints (up to 4 atoms in size) and computing the Tanimoto coefficient, which gives an indication of structural similarity. Most similar compounds are listed first (from top left to bottom right) in the app.

### Predictions targets for new compounds

Molecules active against *Mtb* were identified in recent publications from different groups, these included HTS. Proposed targets for some of these molecules were also identified in these publications, in some cases. These molecules were used as a demonstration set to illustrate use of the app. First they were all drawn in the MMDS app and copied into the TB Mobile app (an example of app-to-app communication). Molecules can also be drawn within the TB Mobile app itself. The similarity searching component was used to rank the content in TB Mobile of molecules with known targets. We have used this as an example of inferring potential targets and compared this to the published data for these molecules. It should be noted that such data is far from definitive as these published compounds have not been tested versus all *Mtb* targets and it is possible the same compound may be active against more than one target.

## Results

### Dataset curation

Over 700 molecules with target related information from the literature were curated for use in CDD and TB Mobile.

### TB Mobile

**Figure 1:**
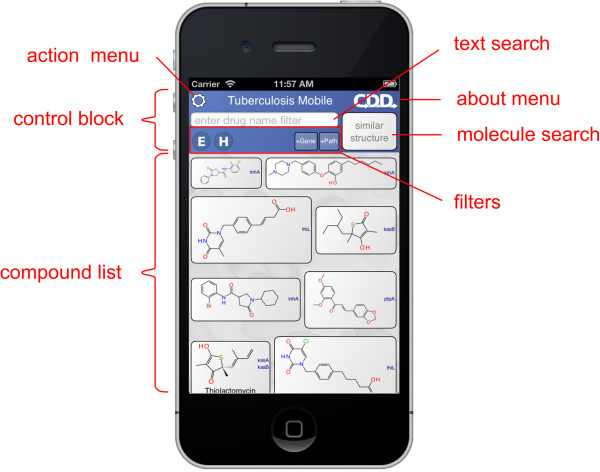
**Appearance of the TB Mobile app and identification of functions on an iPhone. **The appearance of TB Mobile on an Android Tablet.

**Figure 2:**
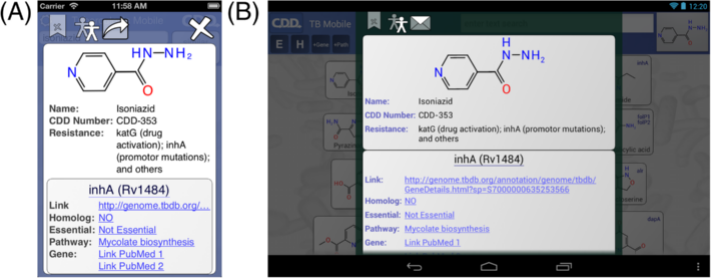
**A. Molecule detail and links on the TB Mobile app on an iPhone. B. Molecule detail and links on the TB Mobile app on an Android Tablet.**

**Figure 3:**
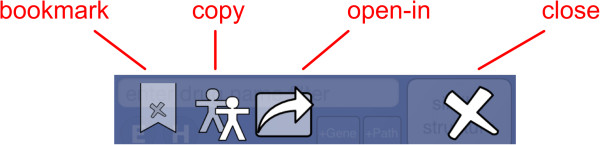
App details in the TB Mobile app on an iPhone.

**Figure 4:**
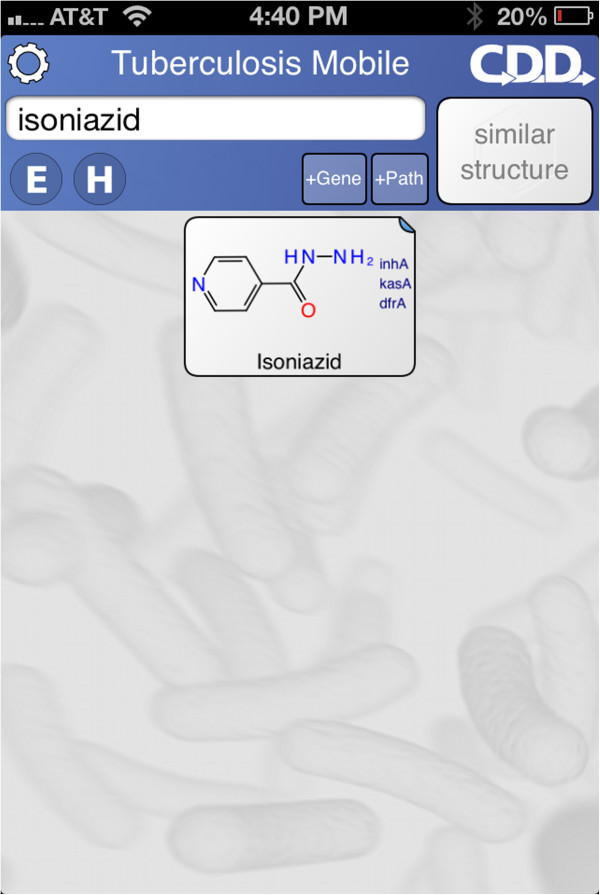
Searching for a molecule in the TB Mobile app on an iPhone.

**Figure 5:**
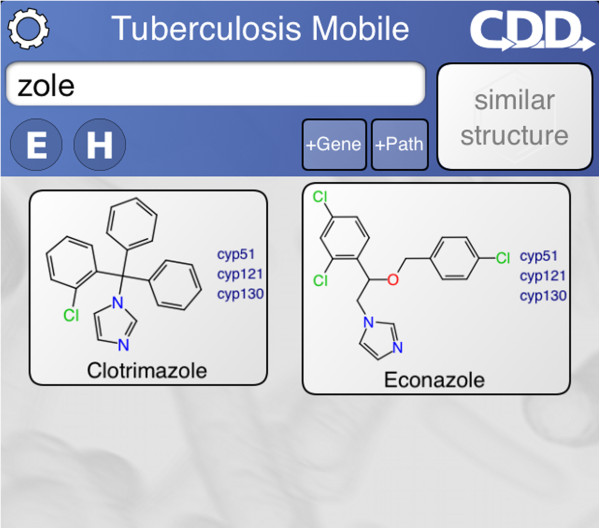
Searching by name in the TB Mobile app on an iPhone.

When the app first opens, it takes a moment to organize its data, then displays the main screen (Figure 1). The screen is divided into two blocks: the control block on top, and the compound list underneath. The control block provides means for searching, sorting and filtering the compound list, as well as access to menus, which will be discussed below. The compound list is a vertically scrollable list of compounds, which are indicated primarily by structure, and annotated by name (if available) and target codes. Tapping on any of the compound buttons brings up the corresponding detail view (Figure 2). Most of the detail view is composed of a scrollable list, which shows all of the available information about the compound. In the above example, the structure of *isoniazid* is shown at the top, along with its name, CDD number, and resistance information. Each of the known target interactions is summarized, which in this case includes InhA. For each target, a variety of information is shown, including human homolog information, whether the target interaction is essential for activity, known biochemical pathways, and a number of links to available reference information. The links can be clicked on, and will launch the mobile browser, providing a significant amount of further detail. The top of the detail view contains several button icons (Figure 3). The select button toggles the bookmark state for the compound. Bookmarked compounds are annotated on the main screen by a folded top right corner (Figure 4). The ‘copy’ button places a copy of the structure onto the device clipboard, so it can be pasted into other mobile apps, or pasted into the molecular structure search box (described in the next section). The open-in button presents a list of installed apps that are capable of opening molecular structures. If one is selected, then that app is launched and provided with the current structure. The control block provides several ways to modify which compounds are listed, and in what order. Note that if none of these features are activated, then all of the compounds are displayed, and their order is selected randomly. To restrict the list of compounds to those whose names contain a certain search string, or CDD number, enter text in the search box (Figure 5). Now only clotrimazole and econazole are shown. Many of the compounds currently have no common name, and so will be excluded from this search.

**Figure 6:**
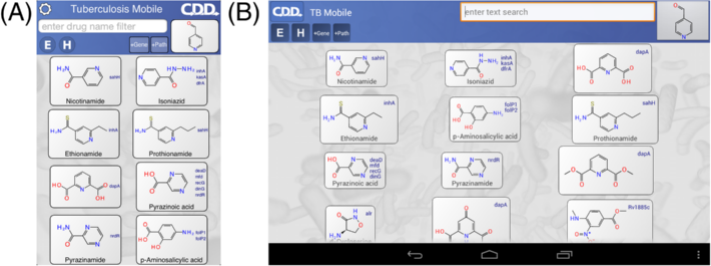
**TB Mobile can be used to rank molecules according to their similarity to a reference structure, which can infer potential targets. A**. an example in iOS version **B**. an example in the Android version.

Structural similarity sorting is accomplished by tapping on the structure box at the top right, which allows a chemical structure to be either pasted from the clipboard, or drawn directly using the built in sketcher. The structure sketcher is based on the powerful gesture-based drawing tools originally developed for the MMDS app, which are designed to make touch-based structure editing fast and effective [40]. For casual users, the app also provides an option to use a less powerful version of the sketcher which is more familiar to users of conventional desktop tools, and so has almost no learning curve. Once the structure is provided, either by editing or pasting, the compound list is sorted according to fingerprint-based similarity to the reference structure (Figure 6). The TB Mobile app can also be opened from other apps that can launch structures (e.g. MolPrime [41]). In this case, the app will perform the structure similarity ordering immediately upon launch.

**Figure 7:**
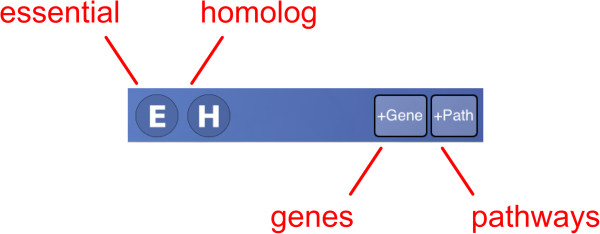
Data can also be filtered by target name, pathway name, essentiality and human ortholog as shown here in the TB Mobile app on an iPhone.

**Figure 8:**
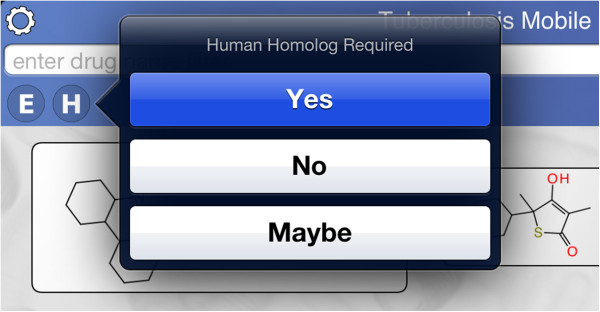
Filtering by homolog in the TB Mobile app on an iPhone.

**Figure 9:**
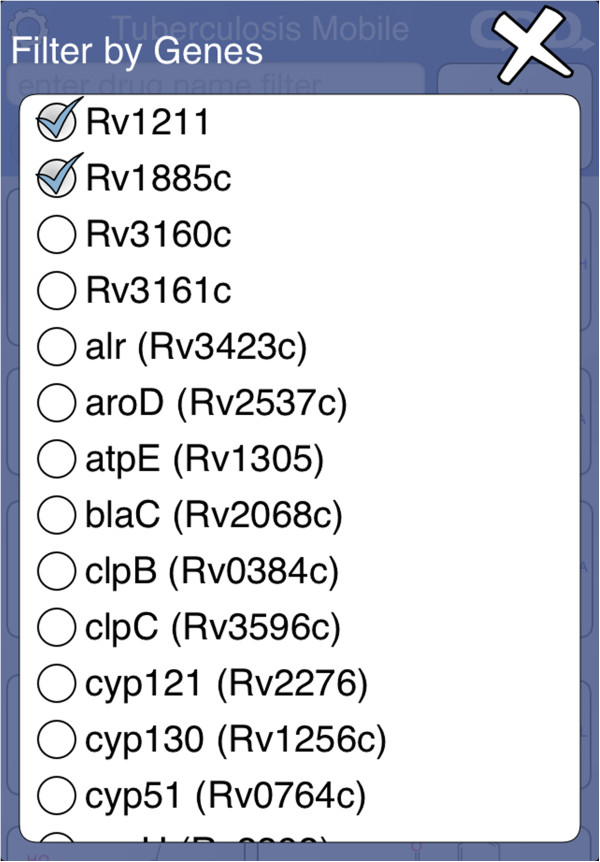
Filtering by genes in the TB Mobile app on an iPhone.

**Figure 10:**
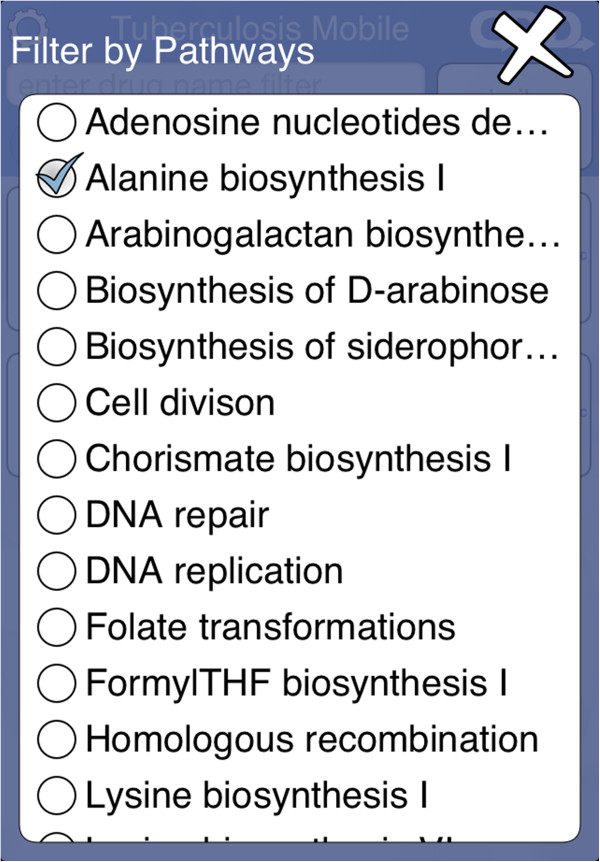
Filtering by pathways in the TB Mobile app on an iPhone.

**Figure 11:**
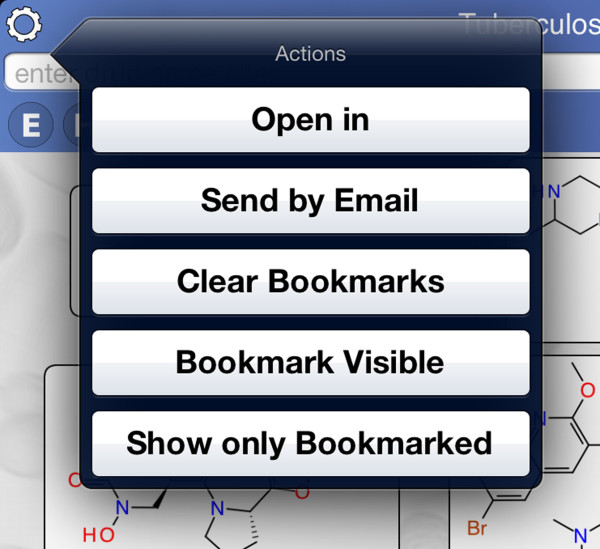
Options for sharing data in the TB Mobile app on an iPhone.

The control block shows several buttons for filtering (Figure 7). The *essential* and *homolog* filters behave similarly: tapping either of these buttons brings up a selection menu with three options (Figure 8). The default state is ‘Maybe’, i.e. no filtering. Selecting ‘Yes’ limits the compound list to only those which have at least one target which is considered essential for activity, or has a human homolog, respectively. Selecting ‘No’ limits the compound list to those which do not have a target satisfying the constraint. The gene filter button brings up a dialog that presents a list of target genes (Figure 9). Each of these genes can be toggled on or off. The compound list will be restricted to those which have activity information about at least one of the selected genes. Similarly, pathways filter button allows known pathways to be selected from an inclusive list (Figure 10). The action menu is activated by pressing the cog icon at the top left (Figure 11). The ‘Open in’ and ‘Send by Email’ actions refer to the compounds that are currently displayed onscreen: these can be bundled into a datasheet and handed off to an app that is capable of handling them (e.g. MMDS, SAR Table [42]), or composed as an outgoing email. Outgoing emails include the compounds as an MDL SDfile attachment. These two techniques make it possible to select a subset of the content in TB Mobile and use it with other apps, or make it available to collaborators. There are several menu actions for handling the bookmarked state: bookmarking all currently listed compounds, viewing only bookmarked compounds, and clearing bookmarks.

### Predicting targets for new compounds

In order to illustrate a workflow for using the mobile app we have curated an additional set of 20 molecules published since 2009 that have activity against *Mtb* and were identified by HTS or other methods (Table 1). In some cases purported targets are known and experimentally verified, while in others a mechanism may be known but a target or targets is unknown. For some, there is no known target and the mechanism is unknown. In each case we have used just the input 2D structure of the molecule in TB Mobile to perform a similarity search of the dataset in the app (Additional file 1: Figures S1-S20 illustrate just the first screen of compounds returned in similarity order to the query molecule). The target for the molecule that is ranked most similar is listed for comparison. Some interesting examples stand-out such as pyridomycin, which was recently shown to target InhA (Rv1484) [43], and TB Mobile was able to rank an InhA inhibitor second (Additional file 1: Figure S12). Gliotoxin was found in a recent HTS screen and resistant mutants could not be generated, so the target was not described [9]. However, this molecule was found to be in TB Mobile (Additional file 1: Figure S13) annotated with the target mycothiol-S-conjugate amidase (Mca, Rv1082) and had been discovered previously [44]. In some cases there are very few molecules which represent inhibitors of a particular target. For example there are only two molecules in TB Mobile that target alanine racemase (Alr, Rv3423c) and two that target dihydrofolate reductase (DfrA, Rv2763c). L2-04 is an example of an inhibitor of Alr identified by HTS [45]. In TB Mobile, a molecule that targets S-adenosylhomocysteine hydrolase (SahH, Rv3248c) is ranked first while the known alr inhibitor, D-cycloserine is ranked 12^th^ out of the set of over 700 compounds (Additional file 1: Figure S1). NC000221 is proposed to target DfrA [46]. Querying TB Mobile with this compound ranked ethambutol as the most similar. Ethambutol targets EmbA (Rv3794), EmbB (Rv3795) and EmbC (Rv3793) and is ranked first while isoniazid (which has many targets including DfrA [47]) was ranked 25^th^ (Additional file 1: Figure S7). This also raises the issue of similarity to compounds like isoniazid that are known to be activated in order to have activity [47]. In other cases there are no representatives of a particular target in TB Mobile e.g. MmpL3 (Rv0206c) [9]. Recently the approved drug oxyphenbutazone (OPB) was identified by HTS as having Mtb activity under aerobic and a 4-stress model of nonreplication [48], although a specific target was not identified. All activity of OPB was shown to be dependent on the acid- and NO dependent conversion of OPB to 4-OH-OPB, which was active on both replicating and non-replicating Mtb. Searching in TB Mobile suggests InhA as a potential target (Additional file 1: Figure S16).

**Table 1:** **Molecules active against *Mtb *evaluated in TB Mobile app**

**Molecule**	**Name**	**Notes from literature**	**TB Mobile results most similar**	**Reference**
	L2-04	Proposed target is Alr	SahH (D-cycloserine ranked 12^th^)	[45]
	L2-05	Proposed target is Alr	SahH	[45]
	L2-06	Proposed target is Alr	SahH	[45]
	L2-10	Proposed target is Alr	DapA	[45]
	L2-12	Proposed target is Alr	SahH	[45]
	L2-13	Proposed target is Alr	ThiL	[45]
	NC00094221	Proposed target is DfrA	EmbA, EmbB, EmbC (Isoniazid ranked 25^th ^(DfrA))	[46]
	DNB1	DprE1/ DprE2	InhA	[49]
	Mirandamycin	Proposed-quinol oxidase	FolP1, FolP2	[50]
	cpd 3	Glyoxylase (rv0577)	FtsZ	[51]
	377790	DprE1	ThiL	[9]
	Pyridomycin	InhA	Def (InhA ranked 2^nd^)	[43]
	Gliotoxin	Not described	Mca (Molecule in database)	[9]
	A039	Glycerol dependent	InhA	[9]
	C215	MmpL3	Cyp51, Cyp121,Cyp130	[9]
	Oxyphenbutazone	none	InhA	[48]
	7759844	Mt-Guab2	InhA	[52]
	GNF-NITD 46	Inhibit ATP homeostasis	Dxs1	[8]
	GNF-NITD 82	Inhibit ATP homeostasis	CysH	[8]
	GNF-NITD101	Inhibit ATP homeostasis	SahH	[8]

### Demonstrating that the app retrieves first line drugs

Fourteen first line drugs active against *Mtb* from a review [53] were used to demonstrate that the molecules are retrieved first and that similar molecules are in the top positions (Table 2). In all cases the query compound is retrieved first. In many cases the second and third molecules are closely related with identical targets (e.g. amikacin, kanamycin and streptomycin). In others the molecules are structurally similar but known targets are different e.g. ethionamide (InhA), prothionamide (SahH) and nicotinamide (SahH). This may be useful for suggesting potential additional targets of compounds for which it is still unclear. An example here is pyrazinamide which is activated to pyrazinoic acid which may have several targets [54,55] (Table 2).

**Table 2:** **First line drugs active against *Mtb *evaluated in TB Mobile app and the top 3 molecules**

**Molecule**	**Name**	**1st**	**2nd**	**3rd**
	Amikacin	Amikacin (FusA1, FusA2, Tuf, EngA, Era)	Kanamycin (FusA1, FusA2, Tuf, RplC, InfB)	Streptomycin (FusA1, FusA2, Tuf, RplC)
	Aminosalicylic acid	Aminosalicylic acid (FolP1, FolP2)	(Rv1885c)	Nicotinamide (SahH)
	Capreomycin 1A	Capreomycin 1A (TlyA)	Capreomycin 1B (RplJ, TlyA)	Capreomycin (RplJ)
	Capreomycin 1B	Capreomycin 1B (RplJ, TlyA)	Capreomycin (TlyA)	Capreomycin (RplJ)
	D-Cycloserine	D-cycloserine (Alr)	Ethambutol (EmbA, EmbB, EmbC)	(DapA)
	Ethambutol	Ethambutol (EmbA, EmbB, EmbC)	(DapA)	D-cycloserine (Alr)
	Ethionamide	Ethionamide (InhA)	Prothionamide (SahH)	Nicotinamide (SahH)
	Gatifloxacin	Gatifloxacin (GyrA)	Moxifloxacin (Murd, PurU)	Ciprofloxacin (GyrA)
	Isoniazid	Isoniazid (InhA, KasA, DfrA)	Nicotinamide (SahH)	Ethionamide (InhA)
	Kanamycin	Kanamycin (FusA1, FusA2, Tuf, RplC, InfB)	Amikacin (FusA1, FusA2, Tuf, EngA, Era)	Streptomycin (FusA1, FusA2, Tuf, RplC)
	Moxifloxacin	Moxifloxacin (Murd, PurU)	(GyrA)	Gatifloxacin (GyrA)
	Pyrazinamide	Pyrazinamide (NedR)	Pyrazinoic acid (deaD, Mfd, RecG, DinG, NedR)	Nicotinamide (SahH)
	Rifampin	Rifampin (RpoB)	Rifapentine (RpoB)	Rifabutin (RpoB)
	Streptomycin	Streptomycin (FusA1, FusA2, Tuf, RplC)	Amikacin (FusA1, FusA2, Tuf, EngA, Era)	Kanamycin (FusA1, FusA2, Tuf, RplC, InfB)

This illustrates they are in the app and that similar molecules with their targets are retrieved.

## Discussion

Within pharmaceutical companies, computational approaches are widely used to aid in drug discovery, but have not been as extensively applied for TB research. We have found several gaps when we look at how computational methods could be used in TB drug discovery including assessing drug-likeness or lead-likeness [56], target deconvolution [35,57], use of sequential virtual and biochemical screening and *in silico* absorption, distribution, metabolism, excretion and toxicity (ADME/Tox) predictions [16]. In the current study we address the target deconvolution issue and methods to assist in prioritization of *Mtb* hits.

In developing mobile apps for cheminformatics we have assumed that many scientists now have a smartphone and/or tablet computer, and that a large majority of these are iOS- or Android-based. Our research aims to deliver cheminformatics solutions via mobile apps as they are easier to use when in the laboratory or in locations without a desktop computer. The TB Mobile app is an example of a reference tool which stores its data locally on the device, so its primary functionality is available even when there is no network connection. While e-lab notebooks are generally used in the office, in contrast a mobile phone will be in the scientists pocket at all times and apps can be used anywhere, anytime and are generally intuitive. Early mobile cheminformatics apps concentrated on solving foundational technical problems like providing a fully functional sketcher on a small, underpowered touchscreen device, and making available computational services [20,21]. TB Mobile is representative of a follow up category which builds on the technical success of the core functionality to provide a very specific product that is highly tuned to the needs of a small but high impact demographic, namely scientists working toward cures for a neglected disease.

We have illustrated a workflow in which compounds derived from *Mtb* HTS or other screening could be input into TB Mobile to perform a similarity search. This enables the user to see if the compound had been previously identified by others (like gliotoxin, Table 1) and also what the most similar molecules are and their known targets (Table 1, Table 2). This could help with potential follow-up and experimental validation. Of course there are examples of molecules that are active *in vitro* due to the experimental conditions and when tested in *in vivo* they have no activity [51], which is an experimental artifact that TB Mobile cannot predict. For some molecules there may be many potential targets in *Mtb*. Even if a molecule has one published target that does not prevent it from having another potential target, it just might not have been experimentally verified. We are not suggesting TB Mobile is a definitive target prediction tool, it certainly is not as sophisticated as other methods that use Bayesian [35] and other methods [57]. It does however set the stage to consider what is possible with a scientific mobile app. While molecular similarity may not be the most accurate method to predict potential targets, it is fast and interpretable. Future methods may use more predictive machine learning models [56,58] but will require expansion of the training set of molecules with targets. We envisage in future that updating the content of the app with molecules and targets not currently represented e.g. adding molecules that target MmpL3 and others should enhance the utility of TB Mobile as well as balance out the heavy bias towards targets that are over represented and which occur more frequently in the results (e.g. InhA). One could also consider addition of some weighting or scoring that would normalize the similarity search for the frequency of a target in the dataset. To date there are representatives of 68 targets that likely are the most important targets over the past decade. We hope to add compounds that represent inhibitors of newer targets in future updates.

## Conclusion

In summary, TB Mobile is a simple to use app with useful functionality for viewing and manipulating data about compounds with activity against *Mtb*, their targets and other related information. The app represents a significant development in the effort to make accessible drug discovery data freely available in a form that is highly useful to scientists in general, not just cheminformatics experts. The mobile app is freely available for iOS (iPhone, iPod, iPad) and Android devices and will be updated regularly.

## Competing interests

Sean Ekins is a consultant for Collaborative Drug Discovery Inc. Alex M. Clark is the founder of Molecular Materials Informatics, Inc., and developed all the apps described.

## Authors’ contributions

SE came up with the idea for TB Mobile, performed all the validation experiments described and wrote the manuscript. AMC developed TB Mobile and wrote the manuscript. MS curated database links to molecules used in TB Mobile and wrote the manuscript. All authors read and approved the final manuscript.

## Supplementary Material

Additional file 1 **The results of the similarity searches for compounds in Table 1 are shown in Additional file 1: Figures S1-S20. **The TB Mobile app is freely available from the Apple iTunes AppStore [25] and Google Play [26]. Click here for file
