# Supplementary material for: TB Mobile: a mobile app for anti-tuberculosis molecules with known targets
Source: J Cheminform. 2013 Mar 6;5:13. doi: 10.1186/1758-2946-5-13 (PMC3616884; doi:10.1186/1758-2946-5-13)

**Supplementary Figures**

**TB Mobile: A Mobile App for Anti-tuberculosis Molecules with Known Targets**

Sean Ekins^1, 2^, Alex M. Clark^3^ and Malabika Sarker^4^

^1^Collaborative Drug Discovery, 1633 Bayshore Highway, Suite 342, Burlingame, CA 94010, USA.

^2^Collaborations in Chemistry, 5616 Hilltop Needmore Road, Fuquay-Varina, NC 27526, USA.

^3^ Molecular Materials Informatics, 1900 St. Jacques #302, Montreal, Quebec, Canada H3J 2S1.

^4^SRI International, 333 Ravenswood Avenue, Menlo Park, CA 94025, USA.

^*^To whom correspondence should be addressed. (e-mail: ekinssean@yahoo.com)

Figure S1. L2-04 similarity search in the TB Mobile app on an iPad. Most similar compounds are listed first (from left to right) in the app.


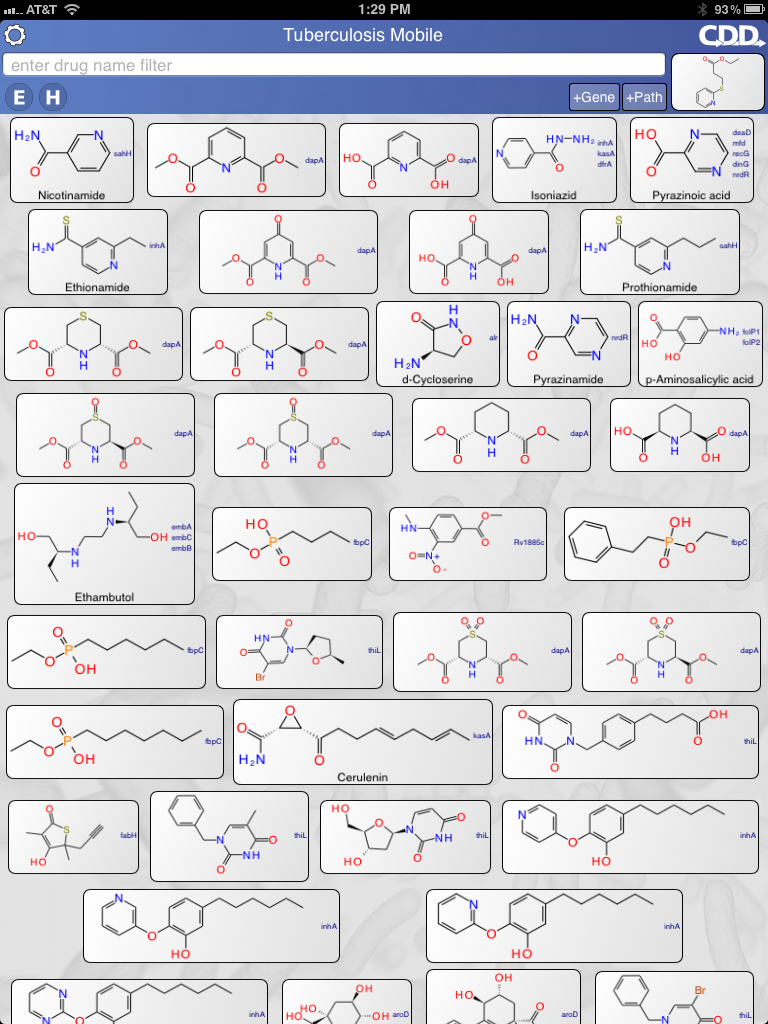


Figure S2 L2-05 similarity search in the TB Mobile app on an iPad. Most similar compounds are listed first (from left to right) in the app.


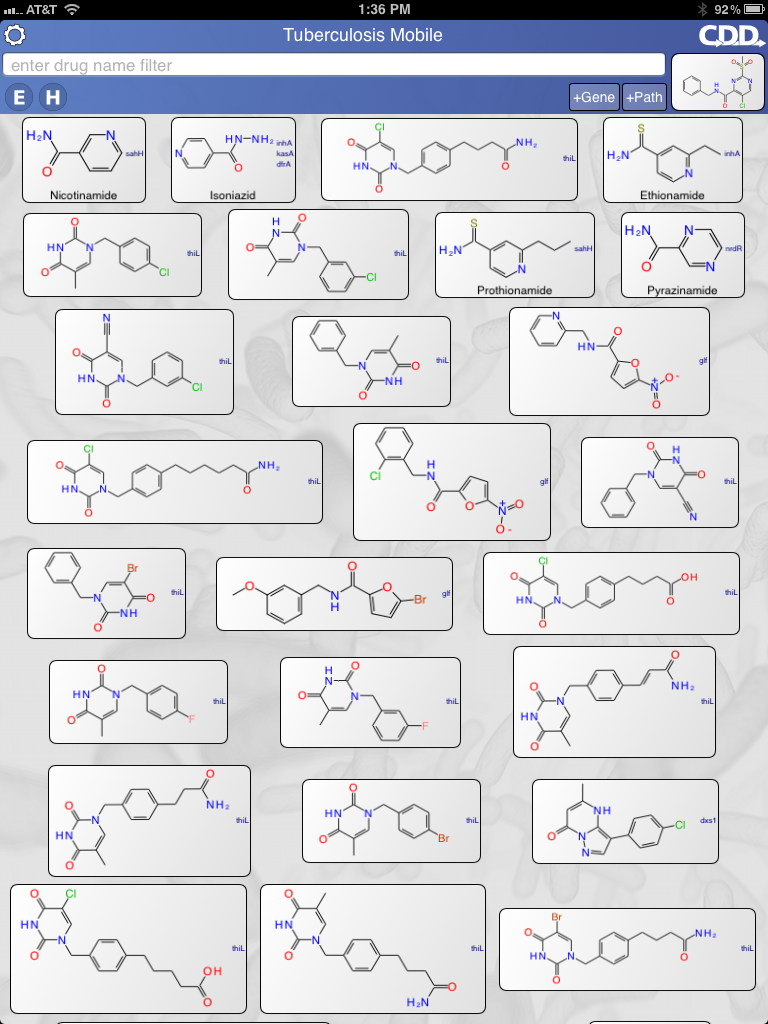


Figure S3 L2-06 similarity search in the TB Mobile app on an iPad. Most similar compounds are listed first (from left to right) in the app.


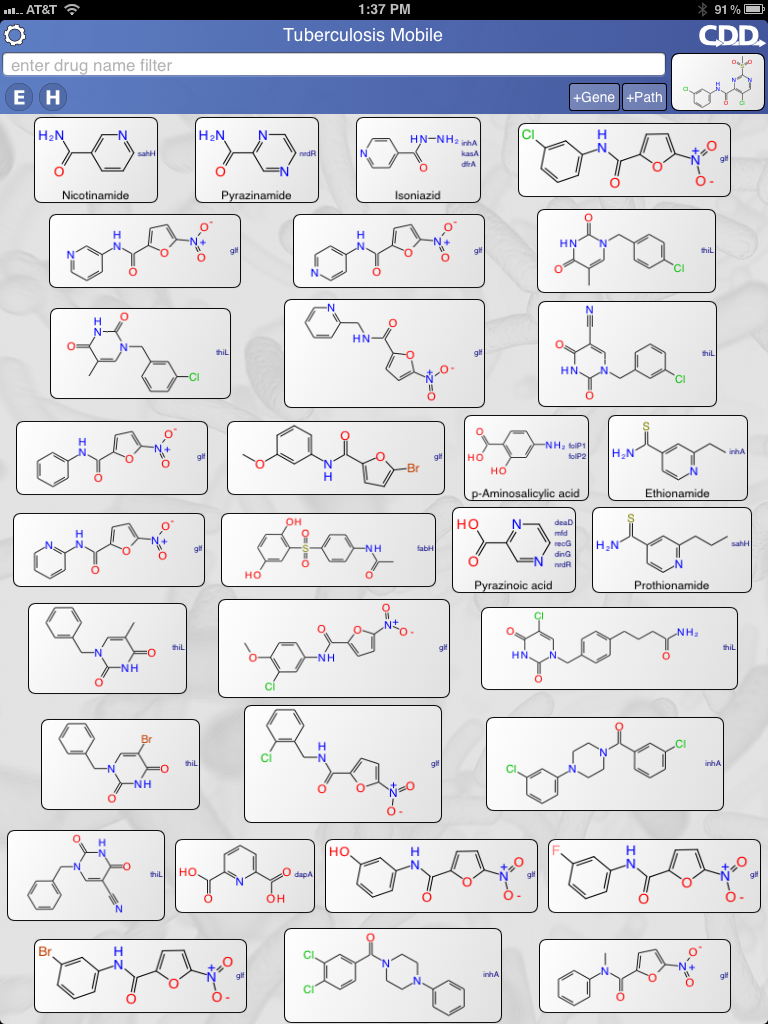


Figure S4 L2-10 similarity search in the TB Mobile app on an iPad. Most similar compounds are listed first (from left to right) in the app.


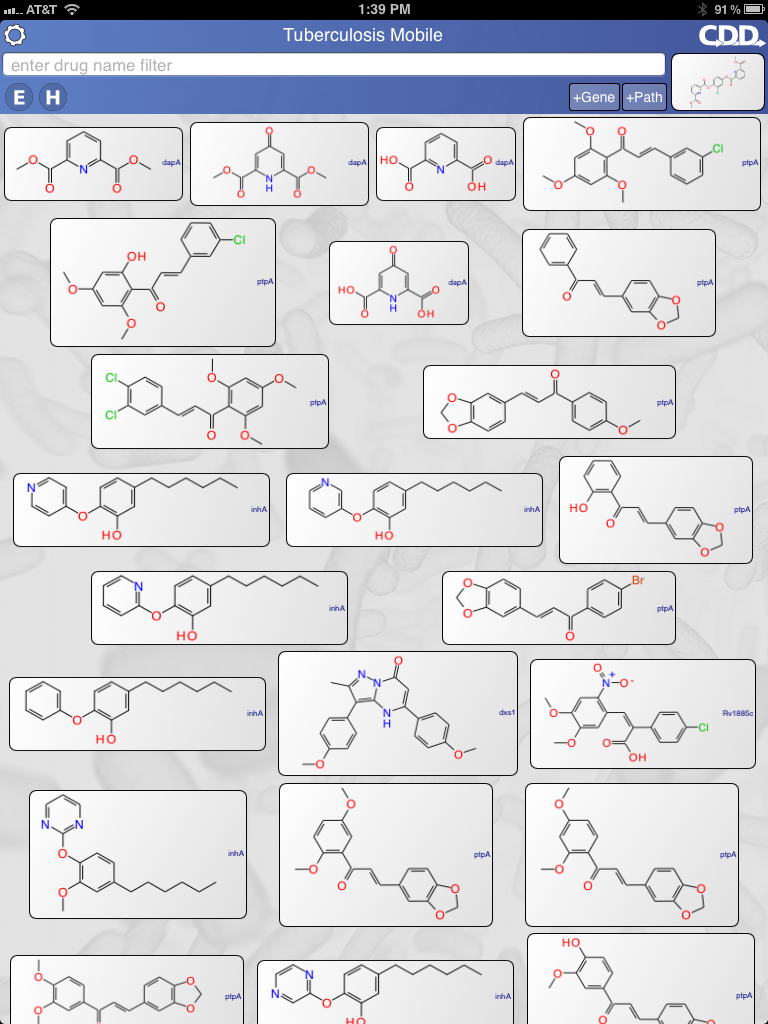


Figure S5 L2-12 similarity search in the TB Mobile app on an iPad. Most similar compounds are listed first (from left to right) in the app.


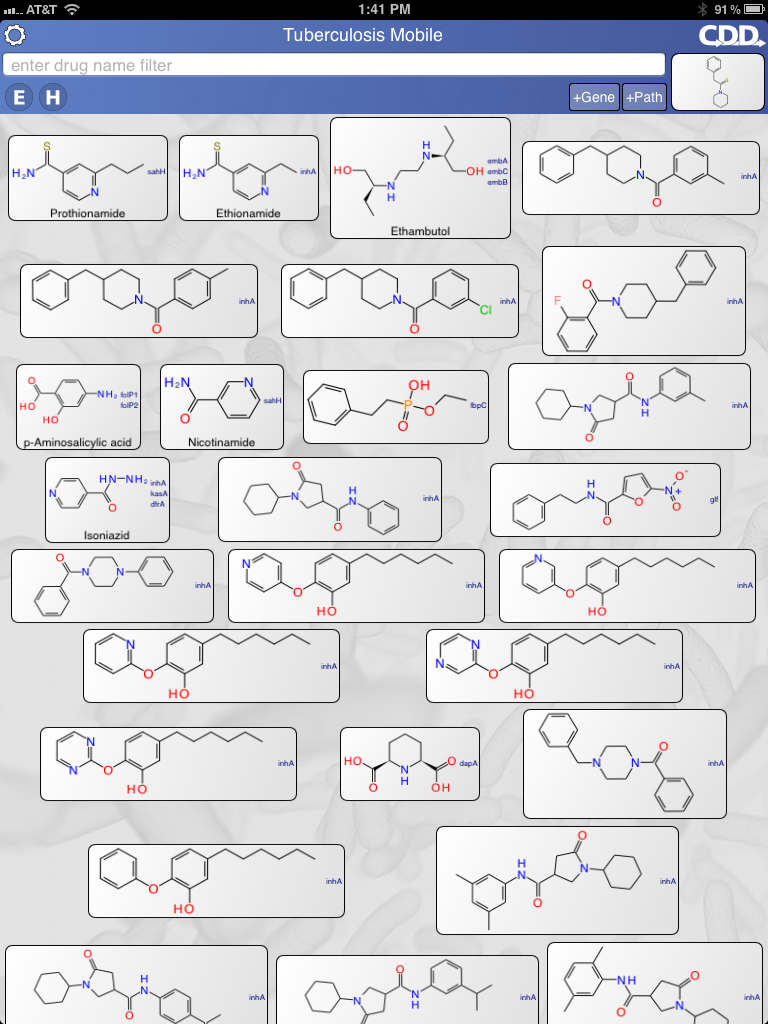


Figure S6 L2-13 similarity search in the TB Mobile app on an iPad. Most similar compounds are listed first (from left to right) in the app.


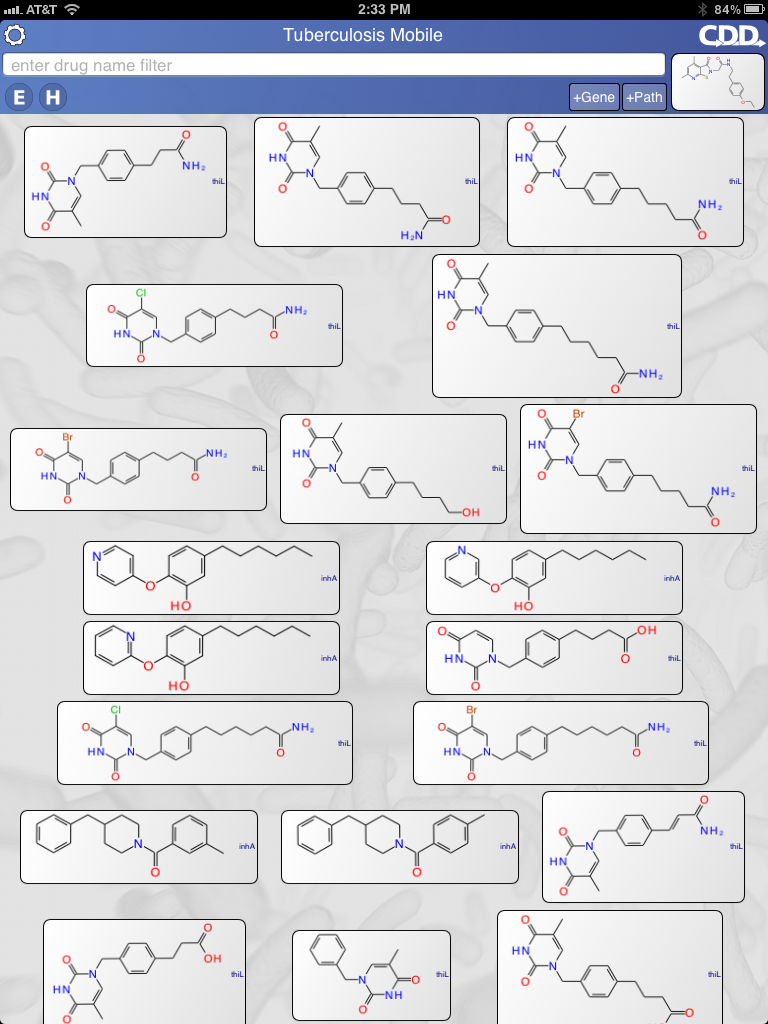


Figure S7 NC00094221 similarity search in the TB Mobile app on an iPad. Most similar compounds are listed first (from left to right) in the app.


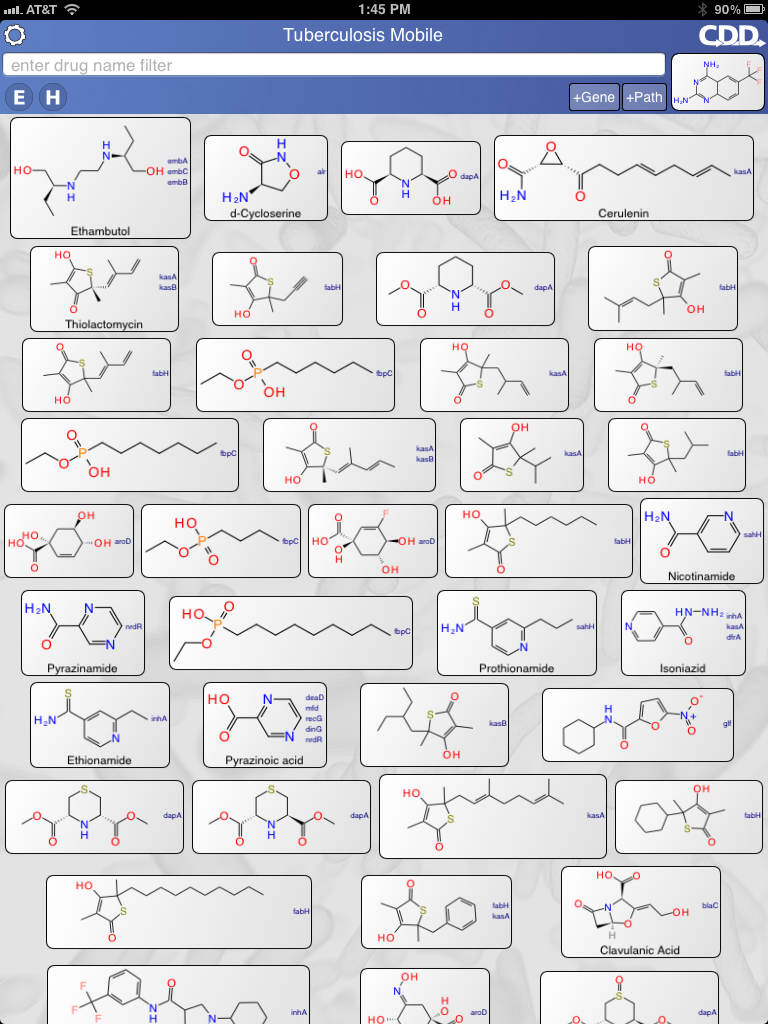


Figure S8. DNB1 similarity search in the TB Mobile app on an iPad. Most similar compounds are listed first (from left to right) in the app.


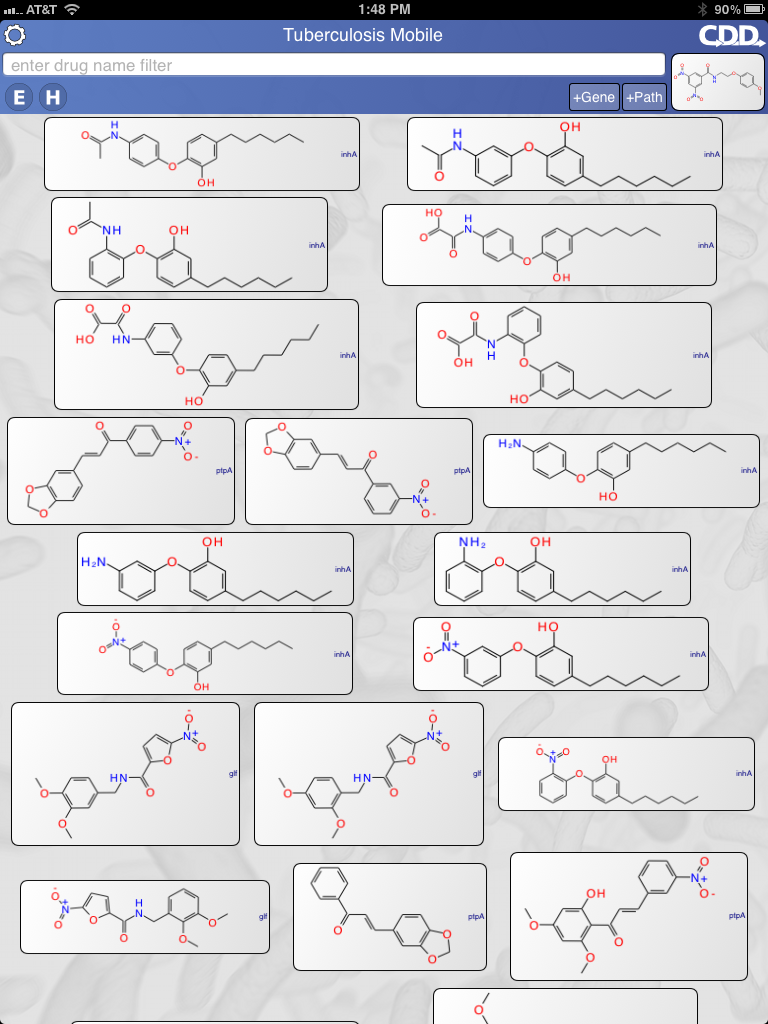


Figure S9 Mirandamycin similarity search in the TB Mobile app on an iPad. Most similar compounds are listed first (from left to right) in the app.


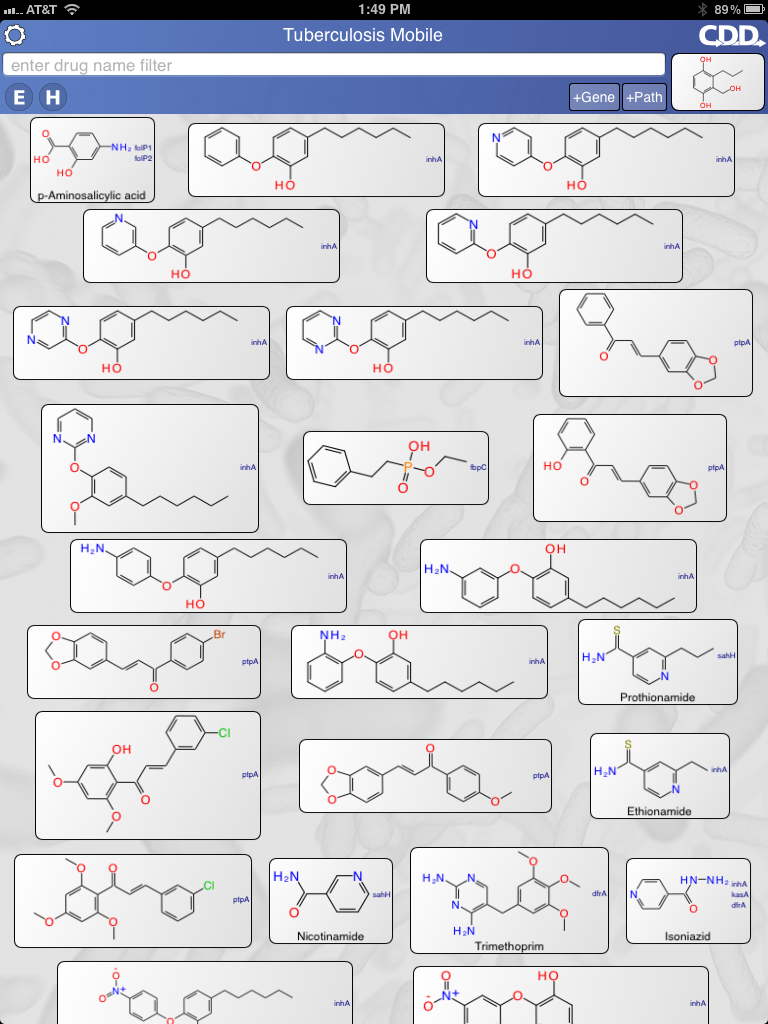


Figure S10 cpd3 similarity search in the TB Mobile app on an iPad. Most similar compounds are listed first (from left to right) in the app.


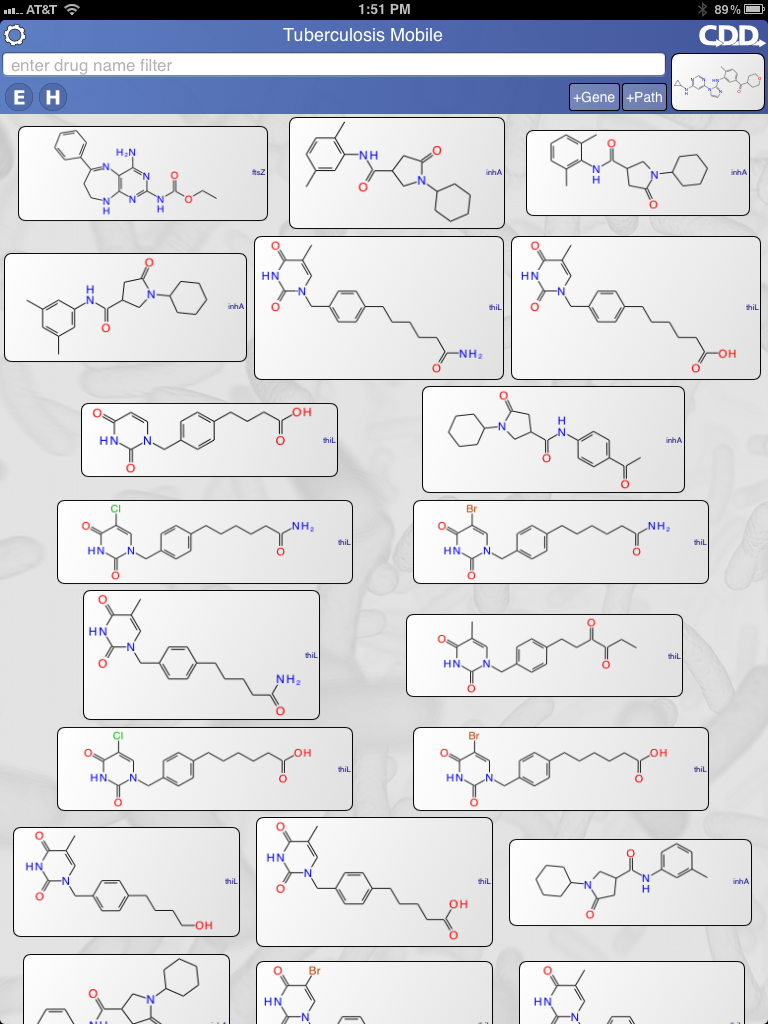


Figure S11 377790 similarity search in the TB Mobile app on an iPad. Most similar compounds are listed first (from left to right) in the app.


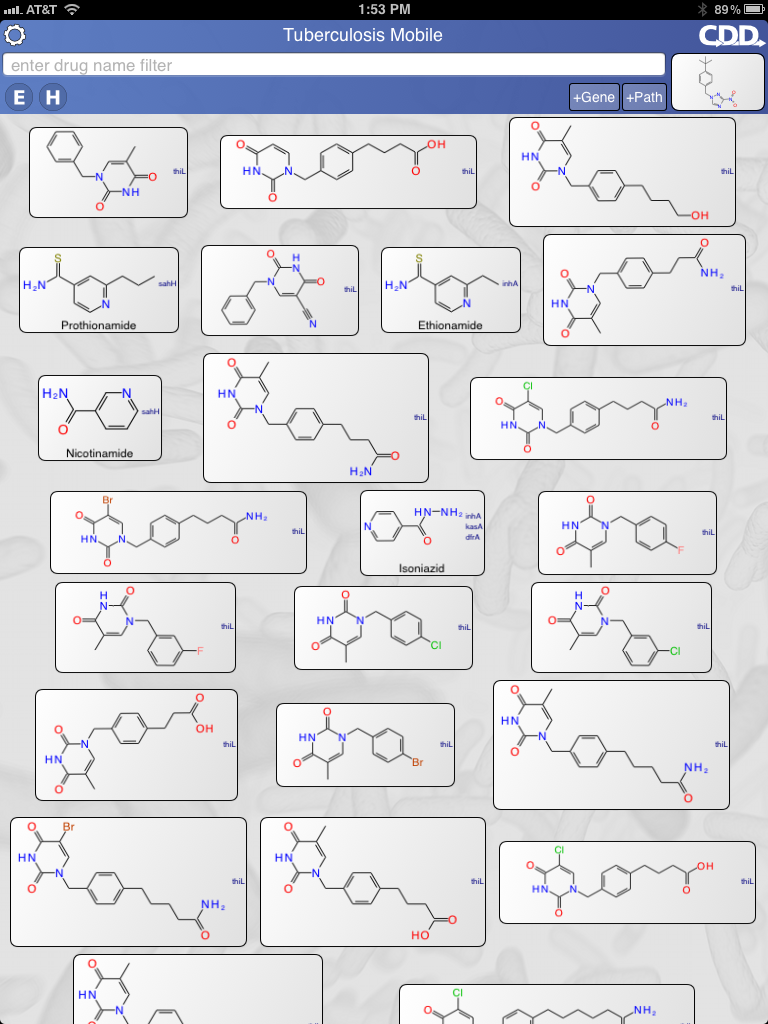


Figure S12 Pyridomycin similarity search in the TB Mobile app on an iPad. Most similar compounds are listed first (from left to right) in the app.


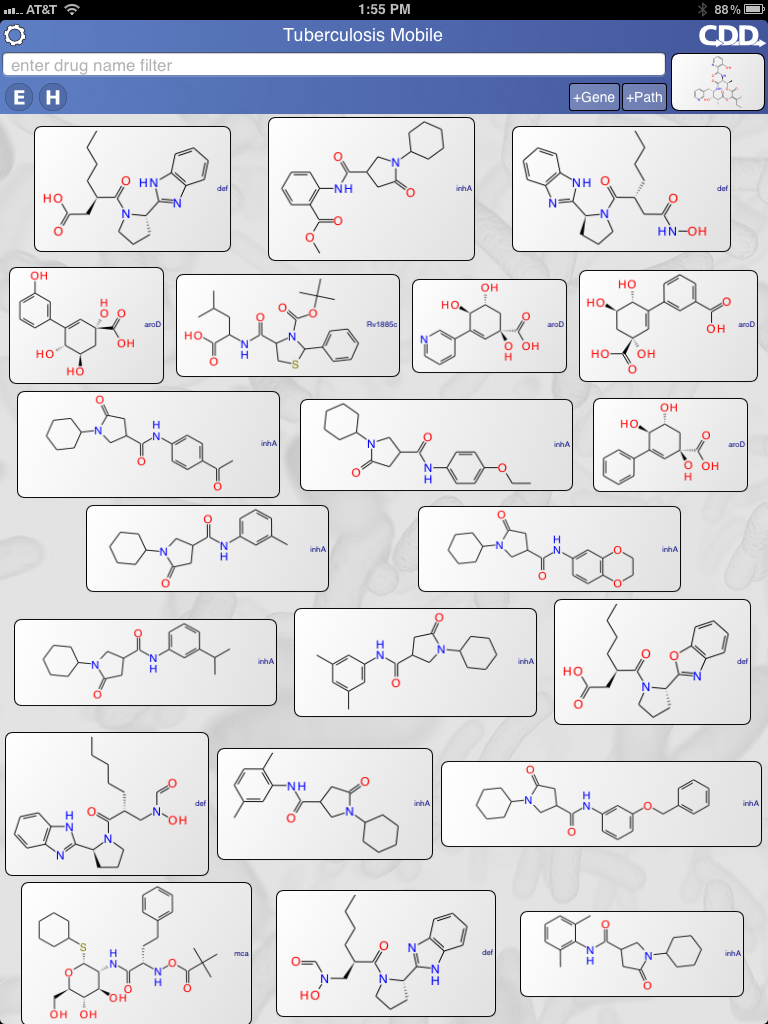


Figure S13. Gliotoxin similarity search in the TB Mobile app on an iPad. Most similar compounds are listed first (from left to right) in the app.


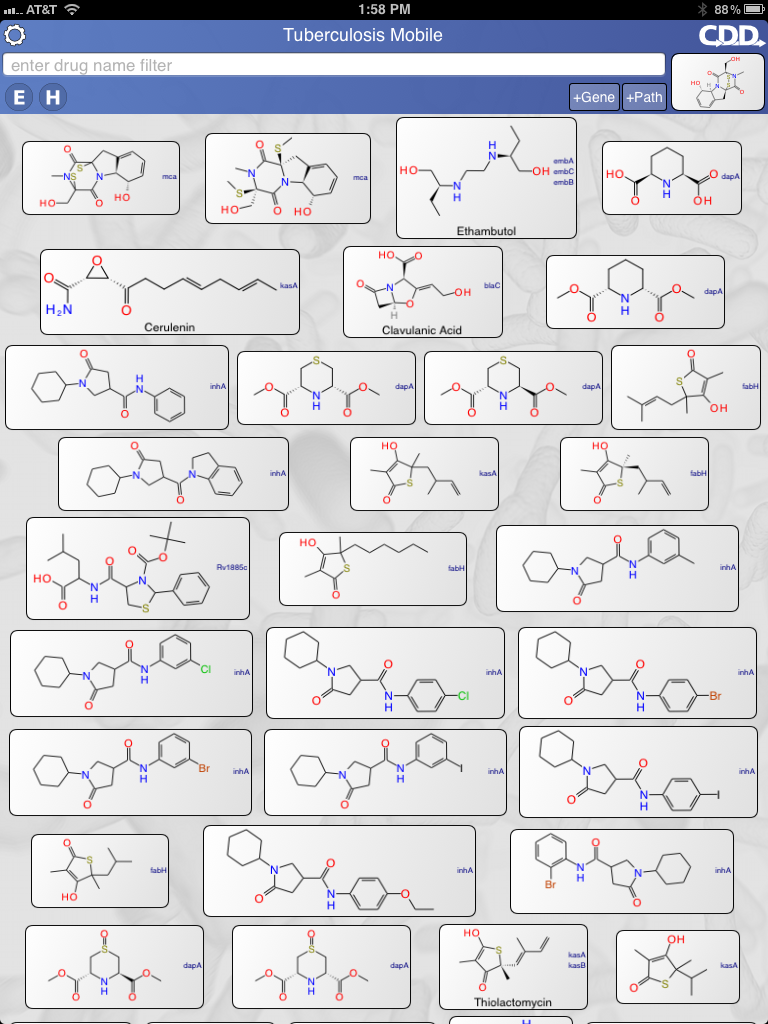


Figure S14 A039 similarity search in the TB Mobile app on an iPad. Most similar compounds are listed first (from left to right) in the app.


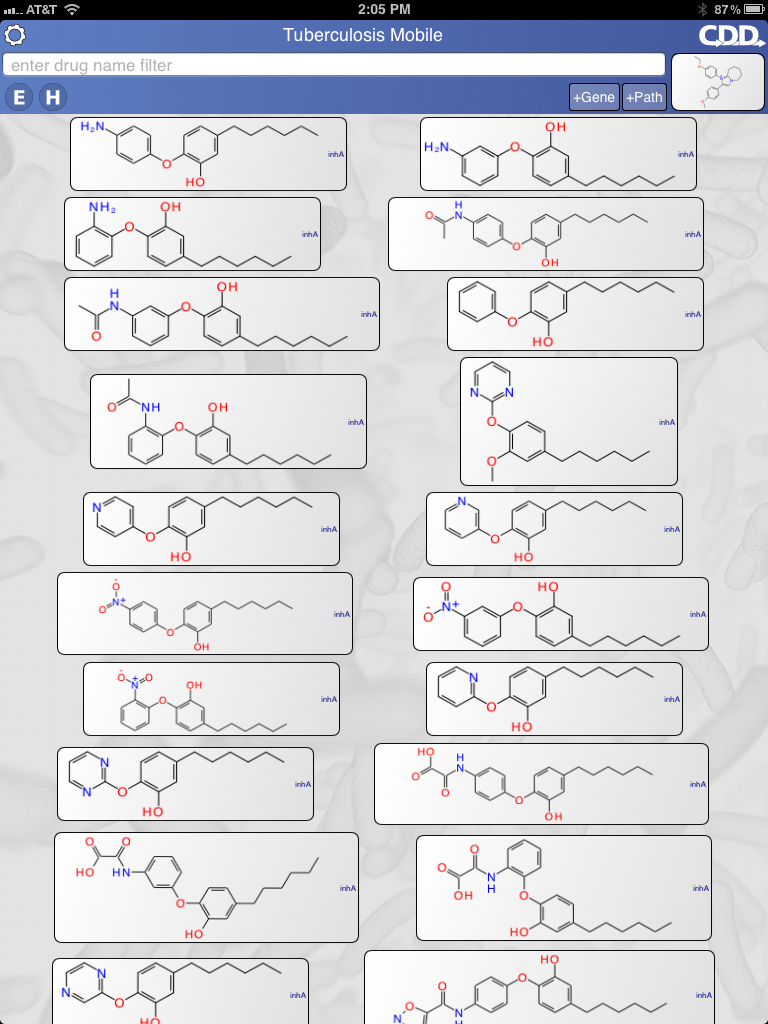


Figure S15. C215 similarity search in the TB Mobile app on an iPad. Most similar compounds are listed first (from left to right) in the app.


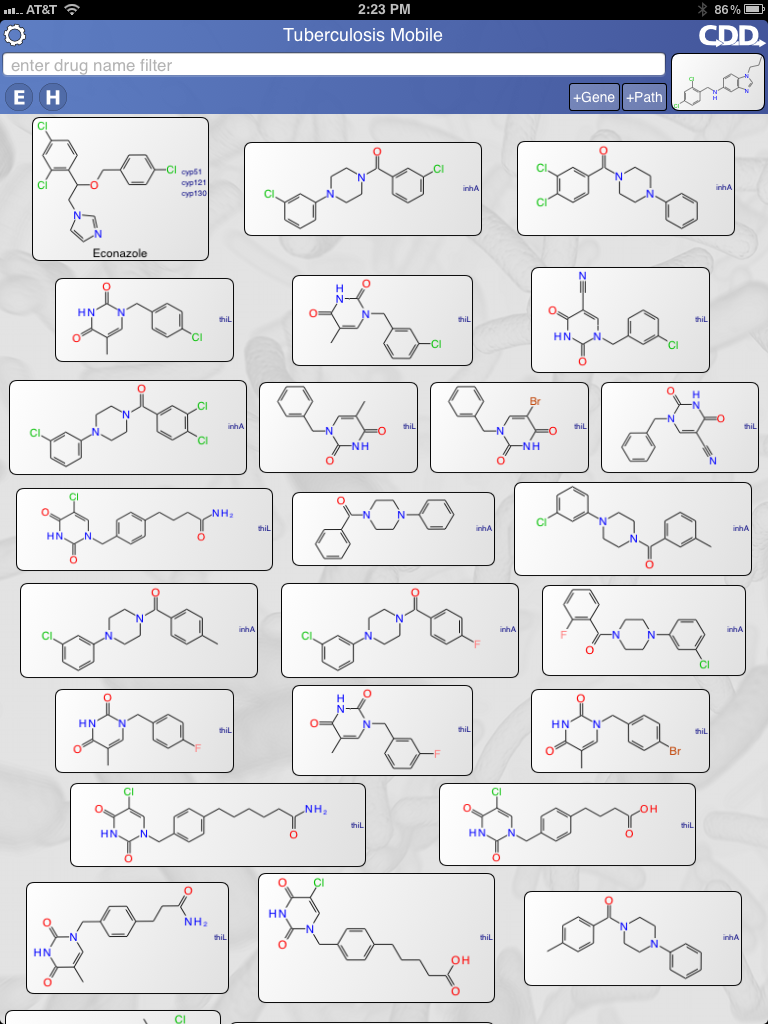


Figure S16 Oxyphenbutazone similarity search in the TB Mobile app on an iPad. Most similar compounds are listed first (from left to right) in the app.


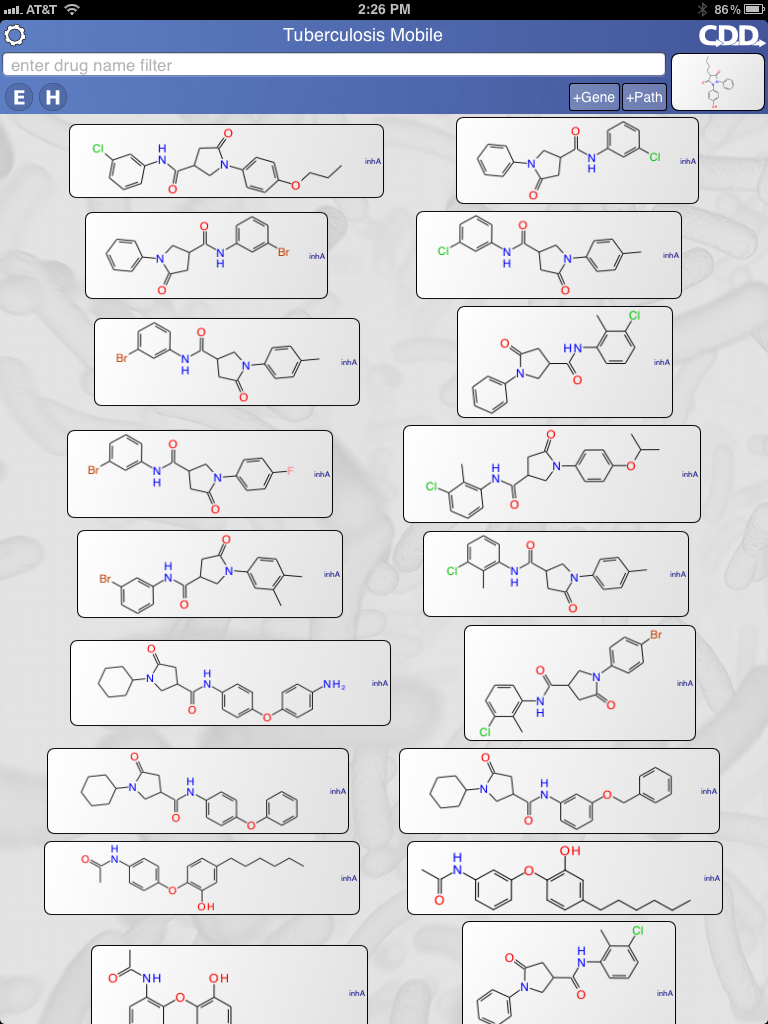


Figure S17 7759844 similarity search in the TB Mobile app on an iPad. Most similar compounds are listed first (from left to right) in the app.


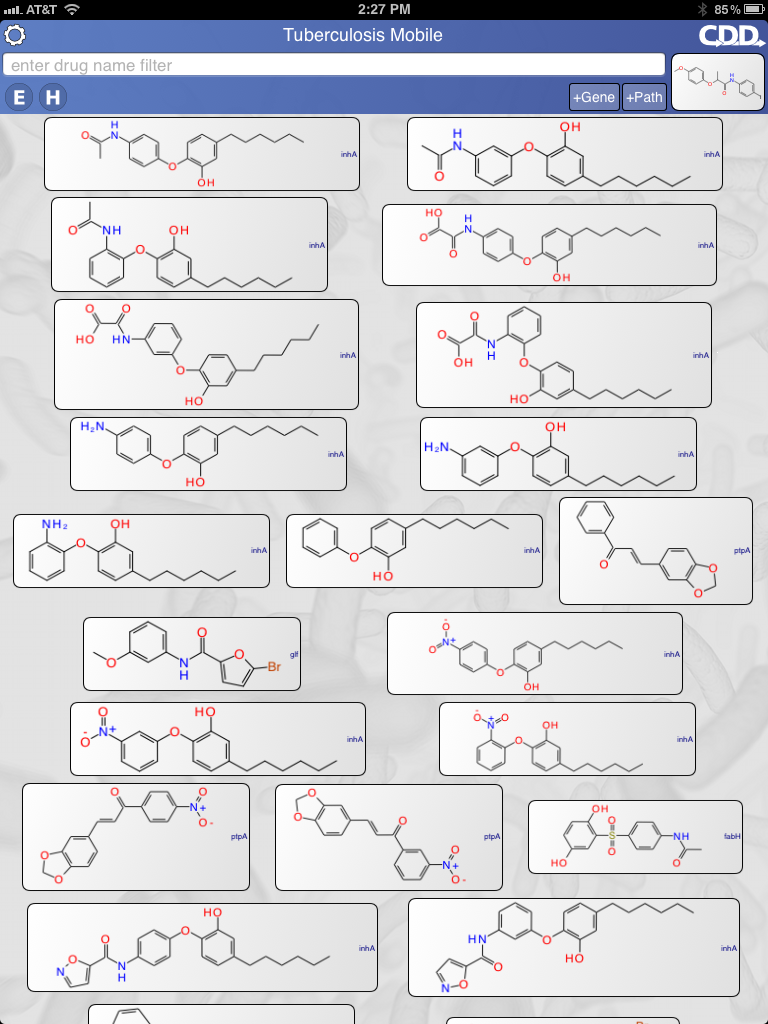


Figure S18 GNF-NITD 46 similarity search in the TB Mobile app on an iPad. Most similar compounds are listed first (from left to right) in the app.


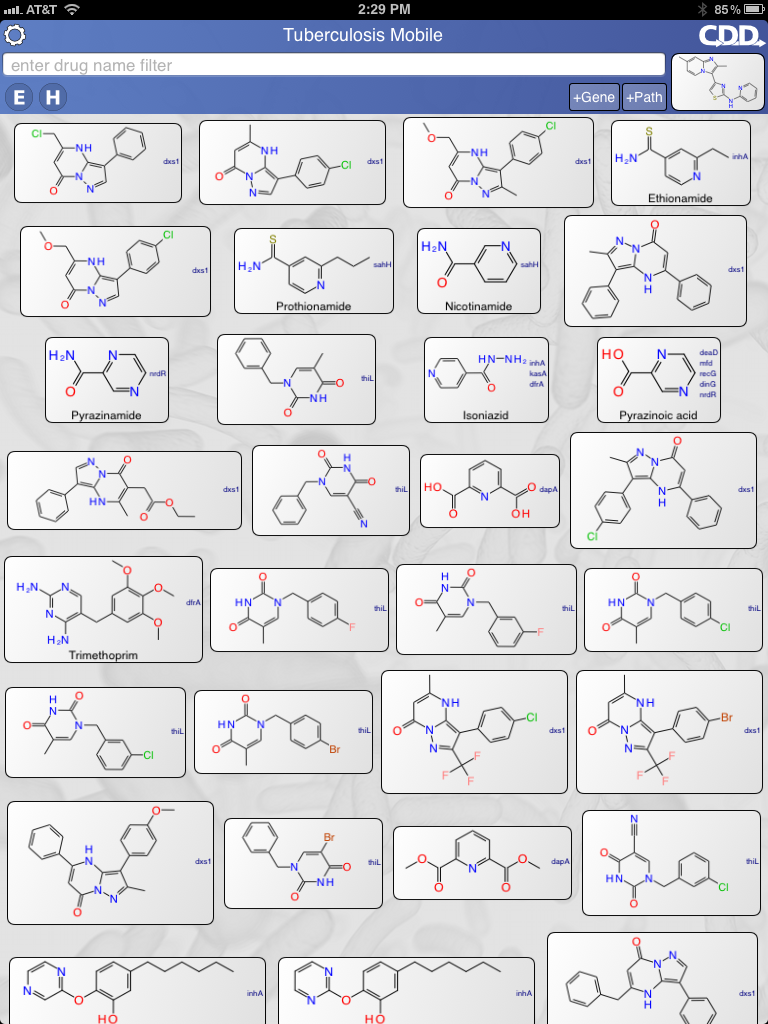


Figure S19 GNF-NITD 82 similarity search in the TB Mobile app on an iPad. Most similar compounds are listed first (from left to right) in the app.


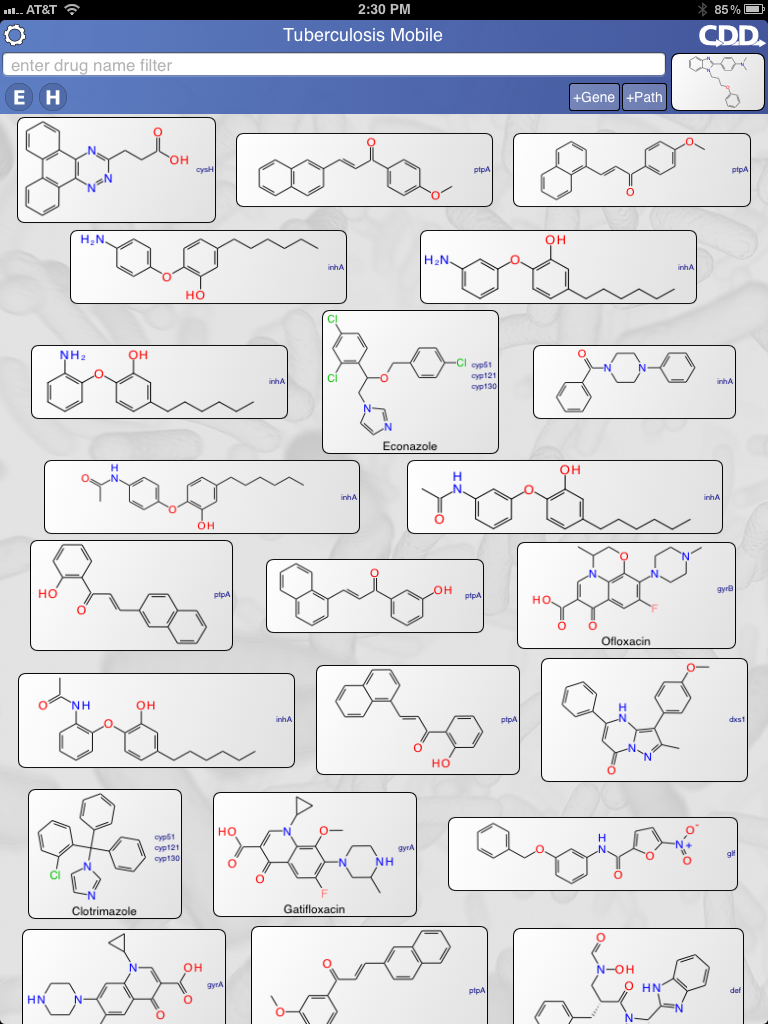


Figure S20 GNF-NITD 101 similarity search in the TB Mobile app on an iPad. Most similar compounds are listed first (from left to right) in the app.


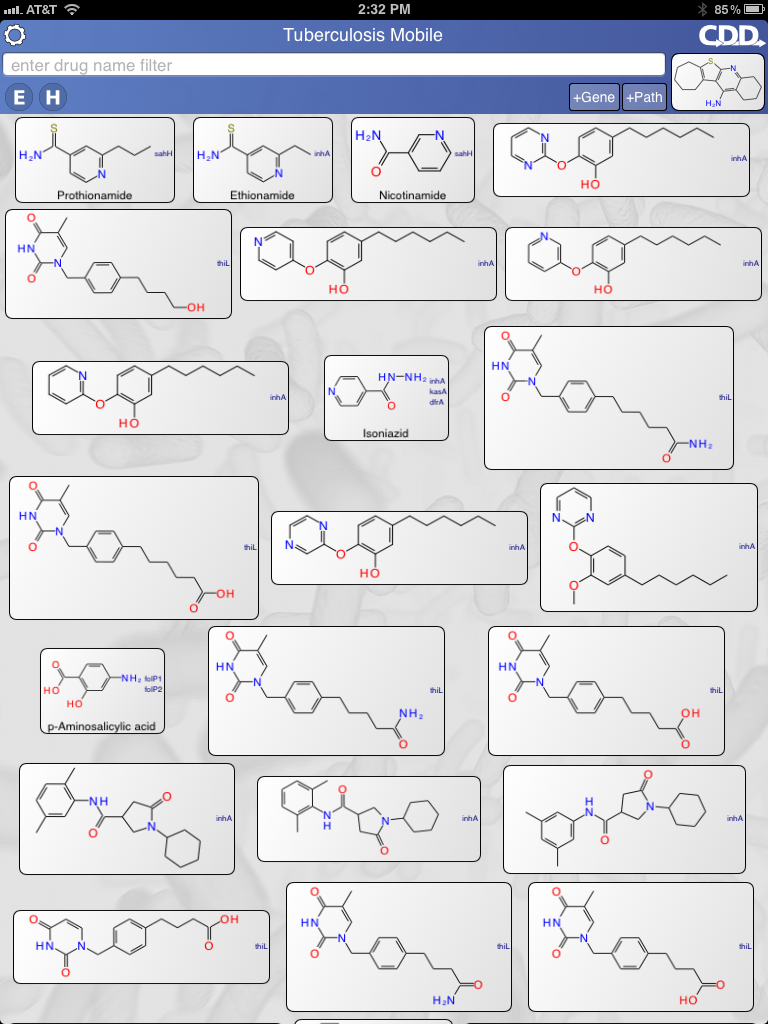

Supplement: Additional file 1 — The results of the similarity searches for compounds in Table 1 are shown in Additional file 1: Figures S1-S20. The TB Mobile app is freely available from the Apple iTunes AppStore [25] and Google Play [26]. [file 1758-2946-5-13-S1.docx]
